# A case of Gitelman syndrome with homozygous *SLC12A3* deletion presenting with epilepsy

**DOI:** 10.1186/s42494-023-00142-3

**Published:** 2023-11-29

**Authors:** Ying Wang, Wenting Huang, Jia Li, Shumin Mao, Wenqiang Fang, Huiqin Xu

**Affiliations:** Department of Neurology, The First Affiliated Hospital of Wenzhou Medical University, Wenzhou, Zhejiang China

**Keywords:** Gitelman syndrome, Epilepsy, Hypokalemia, *SLC12A3*

## Abstract

**Background:**

Gitelman syndrome (GS) is a rare autosomal recessive hereditary renal tubular disorder characterized by hypokalemia, metabolic alkalosis, hypomagnesemia, and hypocalciuria.

**Case presentation:**

We report a rare case of GS with homozygous loss of *SLC12A3* presenting with epilepsy. The patient was a 21-year-old female who sought medical attention for seizures. Her condition primarily manifested as epilepsy, diarrhea, and weakness of limbs. Through genetic analysis, we confirmed the diagnosis of this case and formulated a comprehensive approach for its management.

**Conclusions:**

This case report extends the clinical symptoms of GS and provides a complete family of GS as a reference for subsequent studies.

## Background

Gitelman syndrome (GS), a rare autosomal recessive inherited renal tubular disorder, was first reported by Gitelman et al. in 1966 [1]. This syndrome is defined by impaired reabsorption of sodium chloride in the distal renal tubules, leading to primary hypokalemic metabolic alkalosis, hypomagnesemia, and hypocalciuria [2]. Studies have shown that the incidence of GS-related epilepsy is relatively low (approximately 0.8%) [3]. Previous reports of GS-related epilepsy have been scarce, often lack comprehensive descriptions of patients’ epilepsy symptoms, and often used relatively straightforward diagnostic and treatment approaches [4]. In this case report, we present a case of GS who had exhibited epileptic seizures since birth, with notably prominent epilepsy symptoms. Furthermore, this patient exhibited a notably favorable short-term prognosis. This report was aimed to improve the understanding of GS and elucidate the relationship between epilepsy and this uncommon genetic disorder.

## Case presentation

A 21-year-old female, born in September 2001, presented herself to the Neurology Department outpatient clinic of the First Affiliated Hospital of Wenzhou Medical University due to a history of recurrent limb convulsions and associated consciousness impairments that have persisted for over two decades. The patient had experienced seizure-like episodes since infancy, characterized by symptoms such as hand clenching, eye rolling, teeth clenching, and profuse sweating. These episodes had a frequency of 2-3 times per year and occurred while the patient was awake. In her elementary school years, the seizure episodes progressed from initial visual disturbances characterized by colorful patterns to include symptoms such as blurred vision, cold extremities, trembling hands, and eventual loss of limb control. The patient also experienced episodes of altered consciousness accompanied by severe vomiting, which occurred approximately 6-8 times annually, exclusively at the awake time. Seizures were more likely to be triggered by sudden fright and were more frequent in winter and spring. The episodes were concentrated between 3:00 PM and 6:00 PM. In 2012, the patient sought medical treatment in Fuzhou and was prescribed oxcarbazepine. After 2-3 days of medication, the patient experienced irritability and impulsivity, leading to the self-discontinuation of the medication. After discontinuing the medication, the type and frequency of seizures remained unchanged. In 2019, the patient sought medical treatment in Shanghai and was prescribed levetiracetam (LEV), which effectively controlled the seizures. However, the patient experienced abdominal pain, with diarrhea occurring 6-7 times daily, increased urination frequency, and significant weight loss. After over a year of LEV treatment, the patient decided to discontinue the medication. However, after discontinuing the medication, the patient continued to experience abdominal pain, approximately 2 episodes of daily diarrhea, increased urination frequency, and a change in seizure presentation. The seizure episodes now manifested as generalized stiffness involving the lower limbs, accompanied by vomiting, although the severity of vomiting decreased compared to previous episodes. The patient began to experience limb weakness. In 2023, the patient received treatment at our hospital for epilepsy, diarrhea, and limb weakness. Starting on February 24, 2023, the patient began to take lacosamide (Vimpat) treatment at a dosage of 50 mg bis-in-die (BID). Subsequently, no seizure episodes occurred. The patient’s measurements in February 2023 were as follows: height 158 cm, weight 42 kg, and a body mass index (BMI) 16.8.

### Growth and developmental history

The patient’s growth and development were notably lagging behind her twin sister. Key developmental milestones, such as crawling and walking were delayed compared to her twin sister. The patient exhibited delayed reactions and attained lower academic performance, eventually her education discontinued after completing the ninth grade.

### Past medical history

At the age of 3, the patient experienced a fall from an upper floor, resulting in a half-hour episode of unconsciousness.

### Family history

The patient’s maternal grandmother and maternal great-aunt are sisters, and their marriage involved consanguinity. The patient’s parents are in good health, and she has one twin sister and one brother, both being healthy. The patient’s maternal aunt has a history of epilepsy.

### Laboratory data of the patient

The patient’s laboratory results are presented in Table 1. The patient consistently displayed hypokalemia and hypomagnesemia in examinations. Serum sodium and chloride levels were within the normal range, with other laboratory parameters generally falling within normal limits.

**Table 1:** Laboratory examination of the patient

Parameter	Test value	Reference range
Potassium (mmol/L)	1.83 – 3.48	3.50 – 5.30
Sodium (mmol/L)	138 – 142	137 – 147
Sodium (mmol/L)	94 – 104	99 – 110
Calcium (mmol/L)	2.2	2.10 – 2.60
Phosphorus (mmol/L)	1.14	0.90 – 1.50
Magnesium (mmol/L)	0.42	0.65 – 1.05
pH	7.469	7.350 – 7.450
pO_2_(mmHg)	99.2	83.0 – 108.0
pCO_2_ (mmHg)	44.6	35.0 – 45.0
HCO_3_ act (mmol/L)		22.0 – 26.0
BE (vt) (mmol/L)	7.6	-3.0 – 3.0
Total bilirubin (umol/L)	10	0 – 20
Total protein (g/L)	78.3	65.0 – 85.0
Albumin (g/L)	42.5	40.0 – 55.0
Glucose (mmol/L)	4.2	3.9 – 6.1
Carbamide (mmol/L)	2.1	2.8 – 7.2
Creatinine (umol/L)	71	35 – 80
eGFR	105.2	
Uric Acid (umol/L)	426	155 – 357
Total Cholesterol (mmol/L)	2.86	2.44 – 5.17
Triglyceride (mmol/L)	1.31	0.40 – 1.70
HLD (mmol/L)	0.72	1.29 – 1.55
LDL (mmol/L)	1.95	2.07 – 3.10
Homocysteine (umol/L)	18	0 – 15.0
Creatine kinase (U/L)	88	26 – 140
Thyroxine (nmol/L)	183.36	69.97 – 152.52
TSH (mIU/L)	4.09	0.38 – 5.33
HbA1c (%)	5	4.2 – 6.2
ACTH (ng/L)	32.1	0.00 – 46.0
Cortisol (ng/L)	220.72	
Orthostatic RAAS		
Angiotensin (pg/ml)	98.76	50.00 – 120.00
Aldosterone (pg/ml)	190.6	40 – 310
Renin (pg/ml)	479.1	4 – 38

*eGFR* estimated glomerular filtration rate, *HDL* high-density lipoprotein, *LDL* low-density lipoprotein, *TSH* Thyroid stimulating hormone, *ACTH* adrenocorticotropic hormone, *RAAS* renin–angiotensin–aldosterone system

### Imaging and auxiliary tests

**Fig. 1:**
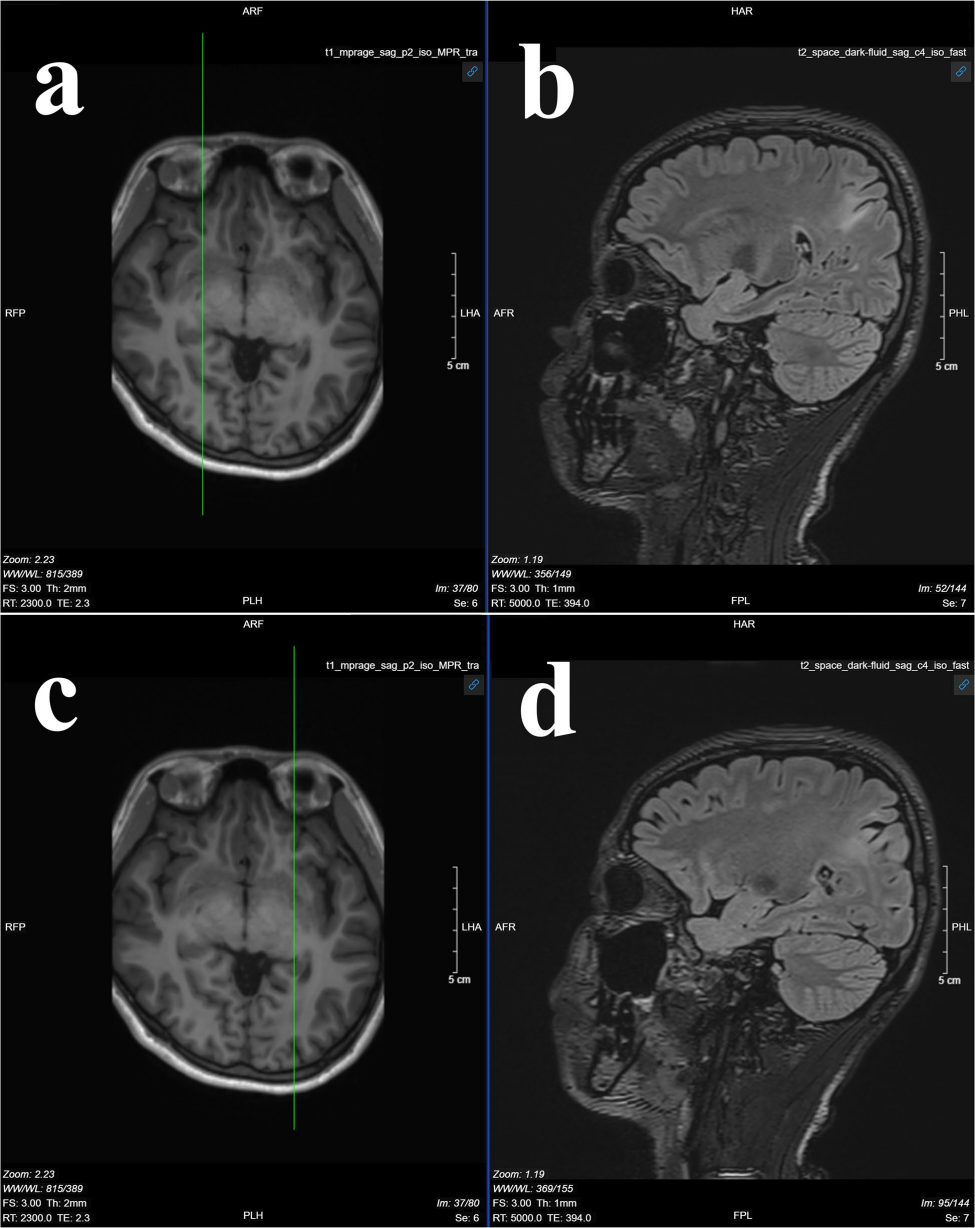
High-resolution hippocampal MRI images showing patchy Flair hyperintensity in the right parietal and left occipital lobes, suggesting the need for contrast-enhanced cranial plain CT scan. Bilateral hippocampal MRI plain scans showed no clear abnormalities. **a** Transverse image. **b** Sagittal image, patchy Flair hyperintensity in the right parietal lobe. **c** Transverse image. **d** Sagittal image, patchy Flair hyperintensity in the left occipital lobe

**Fig. 2:**
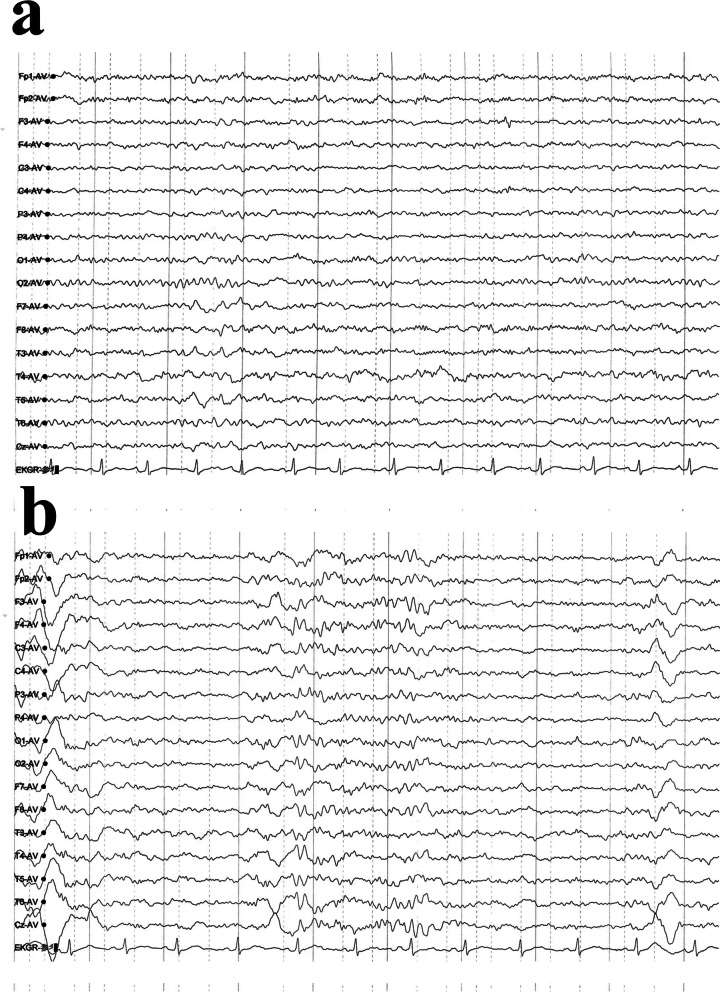
Video EEG recording. **a** Slow waves prominent in the bilateral frontal and temporal regions. **b** Occasional epileptiform discharges observed in the right parietal, posterior temporal, and midline regions during sleep. EEG: electroencephalogram

Cranial plain computerized tomography (CT) scan revealed no obvious brain abnormalities. High-resolution hippocampal magnetic resonance imaging (MRI) showed intermittent Flair hyperintensity in the right parietal and the left occipital lobes (Fig. 1). Video electroencephalogram (EEG) revealed sporadic epileptiform discharges in the right parietal, posterior temporal, and midline regions during sleep (Fig. 2). Electrocardiogram showed sinus rhythm and prolonged QT interval.

### Gene detection

**Fig. 3:**
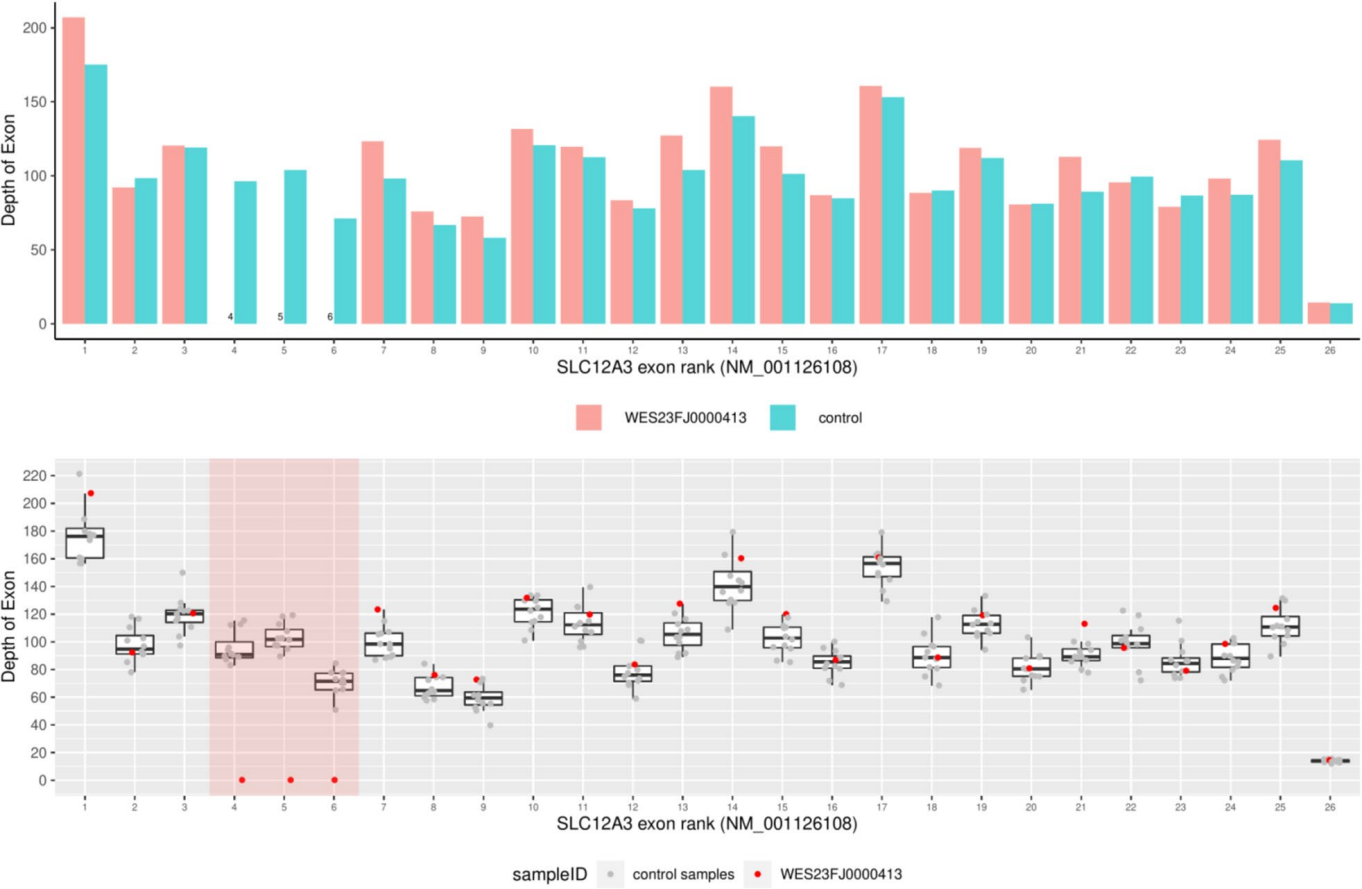
Homozygous deletion of exons 4–6 of the *SLC12A3* gene

After obtaining informed consent from the patient and her family, we collected 3 ml of peripheral blood from the patient and her family members. Subsequently, the collected blood samples were sent to an external testing facility (Xiamen Genokon Medical Technology Co., Ltd.) for comprehensive exome sequencing in the context of genetic disease analysis. The test results indicated a homozygous deletion of exons 4–6 in the *SLC12A3* gene resulting in a copy number (CN) of 0 in the patient (Fig. 3). A mutation of E4_E6del: 1009 bp deletion (c.506_852 del) was identified in this patient. The patient’s parents both carried a heterozygous deletion of exons 4–6 in the *SLC12A3* gene. The patient inherited one pathogenic gene from each parent. This type of genetic deletion, which encompasses the deletion of multiple exons, is rather rare in the general population and holds notable clinical significance. While the variant “*SLC12A3*: exons 4–6 deletion” is not currently listed in the ClinVar database, literature has documented similar exon deletions (exons 4–6 and 5–6) in patients with comparable clinical phenotypes [5, 6]. Thus, in accordance with the pathogenicity classification established by the American College of Medical Genetics and Genomics (ACMG) standards, it has been classified as pathogenic and closely associated with GS [7].

### Diagnosis

The patient presented to our neurology department had a history of recurrent limb convulsions with impaired consciousness for over two decades. Although seizures were triggered by sudden fright in the patient, they also occurred spontaneously. The symptoms during each seizure episode were consistent and not related to specific times or locations. Therefore, psychogenic non-epileptic seizures were not considered [8, 9]. In addition, repeated blood calcium tests consistently showed normal levels, we did not consider low calcium as a cause of seizure-like episodes in the patient. Despite the presence of hypokalemia, the seizures involve not only limb convulsions but also a loss of consciousness. Furthermore, each epileptic seizure experienced by the patient showed an evolving semiology, commencing with visual disturbances and progressing to limb convulsions. The EEG recordings indicate the presence of epileptiform discharges. Hence, we conclude that the patient was indeed experiencing epileptic seizures rather than muscle spasms solely attributable to hypokalemia. Besides, given the patient’s frequent experience of visual symptoms prior to seizures, and based on the MRI and EEG findings, we hypothesized that these seizures originated in the occipital lobe and subsequently evolved into generalized seizures, rather than being absence seizures or myoclonic seizures. Due to the patient’s prolonged history of diarrhea, developmental delay, and epilepsy, whole-exome sequencing (WES) was conducted, revealing an association with GS. The patient’s epileptic seizures were considered symptomatic of a focal onset seizure associated with GS and had a genetic etiology. The diagnosis was GS, with comorbidities of electrolyte disorder, prolonged QT interval, diarrhea, and developmental delay.

## Discussion

The patient reported in this study had suffered seizures since birth, and starting from elementary school, each episode included vomiting. After start of the LEV treatment for epilepsy in 2019, the patient developed frequent diarrhea and increased urination. While stopping the medication alleviated the diarrhea, the symptoms did not fully disappear. Subsequent assessment at our institution confirmed ongoing hypokalemia and hypomagnesemia. Following genetic testing, the patient received a conclusive diagnosis of GS.

The prevalence of GS is estimated to range from 1 to 10 cases per 400,000 individuals, with a potentially higher incidence in Asian populations. Prevalent genetic variations in GS encompass *SLC12A3, NCCT*, *TSC*, and *NCCT* genes, constituting 38%, 2.5%, 2.5%, and 0.8% respectively [3]. *SLC12A3* mutations are the primary cause of GS, as this gene encodes the sodium-chloride cotransporter located in the distal convoluted tubules of the kidney [10]. Currently, more than 500 mutations in the *SLC12A3* gene have been identified (http://www.hgmd.org), with a majority being compound heterozygous. Among them, two gene mutations, p.T60M and p.D486N, are more frequent in the Chinese population, while seven other gene mutations are more prevalent in the European population, p.A313V, c.1180 + 1G > T, p.G741R, p.L859P, p.R861C, c.2883 + 1G > T, and p.C994Y [6, 11–15].

Laboratory investigations of GS typically reveal distinct features such as hypokalemia, metabolic alkalosis, hypomagnesemia, hypocalciuria, and frequent normal or below-average blood pressure levels [2]. In 2019, Muhammad et al. comprehensively reviewed 122 GS cases across 100 articles, creating a table summarizing the clinical presentations and complications of GS patients, as well as the diagnostic and therapeutic approaches. They report that the commonly observed symptoms include fatigue, muscle spasms, vomiting (representing 6%), and polyuria and epilepsy (each contributing 0.8%) [3].

In our patient, EEG analysis revealed evident presence of epileptic seizures. These seizures were not solely attributed to muscle spasms caused by hypocalcemia and hypokalemia induced by GS. A PubMed search using keywords “(GS) AND ((seizure) OR (epilepsy))” yielded 30 articles, indicating that GS does exhibit symptoms of concurrent epilepsy, although the concurrence is relatively rare. Studies suggest that GS may lead to hypomagnesemia (magnesium < 0.7 mmol/L), which could potentially trigger epileptic seizures [4, 16]. This could be a contributing factor to epilepsy in cases of GS. However, further investigation is necessary to gain a comprehensive understanding of the exact mechanisms underlying these epileptic seizures.

Moreover, the patient exhibited prolonged QT intervals, potentially attributed to GS-induced electrolyte imbalances. Moderate to severe hypokalemia and hypomagnesemia, along with mild hypocalcemia, all have the potential to result in QT interval prolongation [17–19]. This prolongation can result in arrhythmias [17]. Therefore, in GS patients with electrolyte disturbances, it is essential to promptly correct these imbalances and consistently monitor blood potassium and magnesium levels.

Prolonged hypokalemia can lead to the formation of renal vacuoles, potentially affecting the renal function of patients [13]. In our case, the creatinine level was 71 μmol/L, which, although is within the normal range, warrants attention due to the patient’s weight (BMI = 16.8) and nutritional status. Therefore, it is crucial to periodically evaluate kidney function during treatment.

**Fig. 4:**
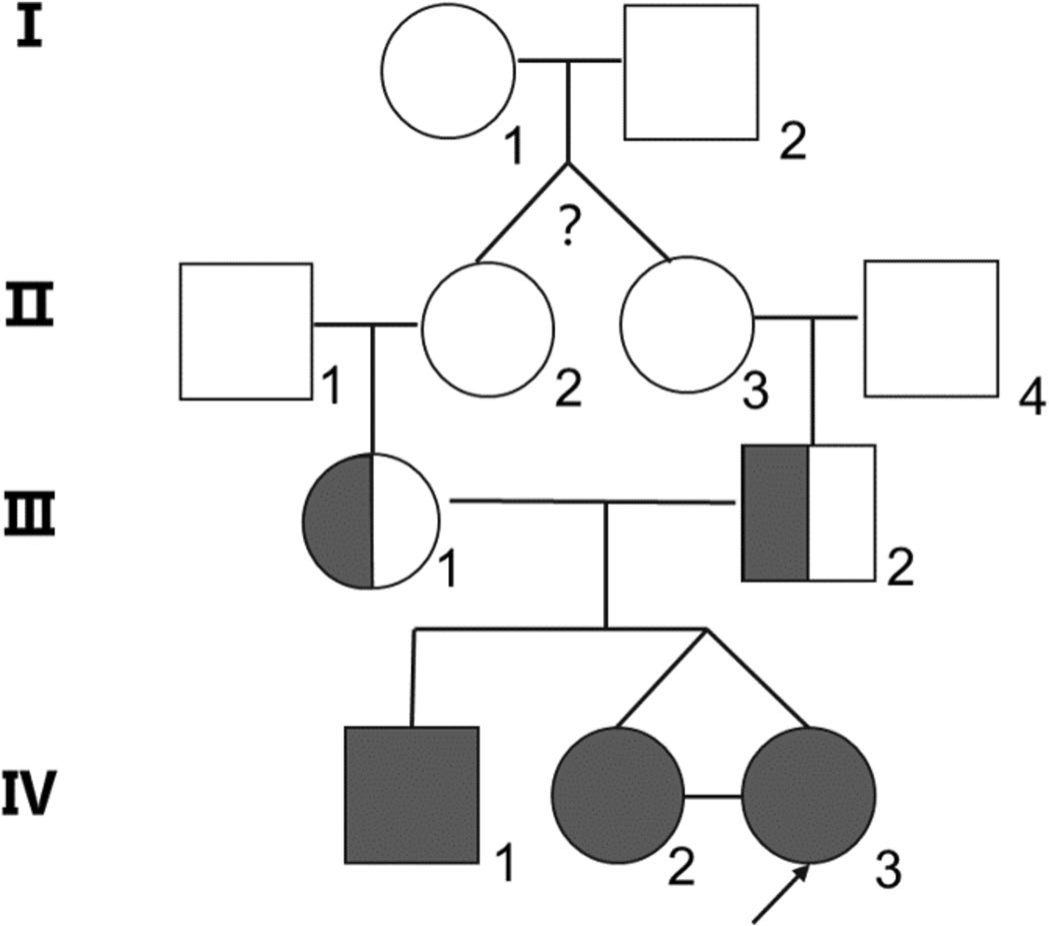
A pedigree chart

In this case, whole-exome sequencing unveiled a homozygous deletion of exons 4–6 (CN = 0) in the *SLC12A3* gene. To confirm this finding, we conducted quantitative polymerase chain reaction on the patient and her parents, twin sister, and brother. The results indicated that the patient’s parents possessed heterozygous variations in *SLC12A3* exons 4–6, while the patient and their siblings exhibited homozygous variations in *SLC12A3* gene exons 4–6 (Fig. 4). Interestingly, the patient’s older brother and sister only exhibited low blood potassium and magnesium levels during electrolyte examinations, without experiencing muscle spasms, developmental delays, seizure, limb weakness or gastrointestinal symptoms such as diarrhea. This implies the existence of clinical diversity within the family. While perplexing, similar cases have been documented in literature. In 2006, Ng et al. reported a GS pedigree where both siblings harbored the same mutation, yet only the brother exhibited symptoms, such as mild to moderate hypokalemia, hypomagnesia, and periodic paralysis commencing at age 11 [20]. This suggests that although GS is an autosomal recessive genetic disorder, its clinical manifestation may be influenced by other factors. Hence, for patient management, it is essential to maintain regular clinical follow-ups of the patient’s siblings, paying special attention to monitoring their potassium levels and administering potassium supplementation when needed.

### Treatment

Currently, there is a lack of a comprehensive and definitive treatment for GS, and GS remains incurable. Treatment primarily involves symptomatic management tailored to the patient’s clinical manifestations. Timely supplementation of potassium and magnesium electrolytes is of paramount importance. Investigation of interventions such as potassium-sparing diuretics, cyclooxygenase inhibitors, and medications like angiotensin-converting enzyme inhibitors or angiotensin receptor blockers may help alleviate the electrolyte imbalances [2, 21, 22].

To optimize the treatment for the patient, a multidisciplinary case discussion was conducted with specialists in gastroenterology, endocrinology, and nephrology. The patient’s primary clinical symptoms encompass epilepsy, diarrhea, polyuria, and limb weakness. Epilepsy was effectively controlled with lacosamide 50 mg BID, with no recent recurrence of seizures. To manage diarrhea, probiotics can be considered for loose stools, and diosmectite for watery stools. The patient exhibited favorable response to such interventions. Research suggests that LEV may cause gastrointestinal side effects, including diarrhea [23]. Despite discontinuation of LEV, the patient’s diarrhea remained a concern. To identify the root cause of the diarrhea, a gastrointestinal endoscopy is recommended after potassium levels are stabilized. Because the patient’s hypokalemia persisted, oral potassium supplementation had limited effectiveness. The treatment was adjusted to include spironolactone and oral potassium magnesium aspartate, along with potassium chloride sustained-release tablets, guided by blood potassium levels. The patient’s blood potassium was reevaluated 1 week after the medication adjustment and, although still below the normal range, it was increased to over 3.0 mmol/L. Therefore, patients are encouraged to include potassium- and magnesium-rich foods such as nuts and grains in their diet.

## Conclusions

This case report adds to the record of homozygous exon 4–6 deletion in the *SLC12A3* gene. It emphasizes the diversity and complexity of GS clinical presentations within a family context, providing insignt for future research. Additionally, this case report expands the clinical manifestations of GS, spotlighting a patient with epilepsy as the primary presenting symptom. While GS is typically first encountered in nephrology, endocrinology, and other medical fields, neurologists should be aware of the possibility of GS when rare seizures constitute the primary clinical presentation.

## Data Availability

The datasets used and/or analysed during the current study are available from the corresponding author on reasonable request.

## Abbreviations

ACMG: American college of medical genetics and genomics
BID: Bis-in-die
BMI: Body mass index
CN: Copy number
CT: Computerized tomography
EEG: Electroencephalogram
GS: Gitelman syndrome
LEV: Levetiracetam
MRI: Magnetic resonance imaging
WES: Whole exome sequencing
